# Allele-specific gene expression in F1 hybrid mice reveals structural variants affecting macrophage characteristics

**DOI:** 10.1038/s41598-025-21643-w

**Published:** 2025-10-29

**Authors:** Keisuke Yoshida, Toyoyuki Takada, Masafumi Muratani, Takanori Amano

**Affiliations:** 1 Present Address: Next Generation Human Disease Model Research Team, RIKEN BioResource Research Center, Tsukuba, Ibaraki 305-0074 Japan; 2 Present Address: Institute for Advanced Medical Sciences, Nippon Medical School, Bunkyo-ku, Tokyo 113-0031 Japan; 3 Present Address: Integrated Bioresource Information Division, RIKEN BioResource Research Center, Tsukuba, Ibaraki 305-0074 Japan; 4 Present Address: Department of Genome Biology, Institute of Medicine, University of Tsukuba, Tsukuba, Ibaraki 305-8575 Japan

**Keywords:** Allelic expression, Mouse strain, Macrophage, Innate immunity, Structural variation, Genetics, Immunology

## Abstract

Genetic diversity underlies the foundation for variations in gene expression, resulting in diverse phenotypic traits. In this study, we investigated the effect of genetic variation on macrophage function using two genetically distinct mouse strains, C57BL/6 (B6) and Japanese Fancy Mouse 1 (JF1), which exhibit substantial genetic polymorphisms. Gene expression analysis of macrophages derived from B6, JF1, and their F1 hybrids revealed strain-specific allelic expression of immune-related and glycolytic genes, indicating significant influence of *cis*-regulatory variants on macrophage function. These findings highlight the role of genetic variation in shaping immune responses and metabolic pathways and provide new insights into the genetic basis of phenotypic diversity in macrophages among subspecies of mice.

**Supplementary Information:**

The online version contains supplementary material available at 10.1038/s41598-025-21643-w.

## Introduction

Innate immunity is a type of immune response that provides immediate and nonspecific protection against various pathogens such as viruses, bacteria, fungi, and parasites^1–3^. Innate immunity is highly conserved across various organisms and serves as the first line of defense against invading pathogens. Unlike adaptive immunity, which involves the production of specific antibodies targeting particular pathogens, innate immunity does not recognize or remember specific pathogens. However, it plays a crucial role in activating and enhancing the adaptive immune response by alerting the body to the presence of pathogens and providing essential signals for initiating the adaptive immune response.

Macrophages play a critical role in the immune response to infection and injury^4^. They can be activated in different ways for performing various functions, with two primary activation states known as M1 and M2^5,6^. M1 macrophages are activated by inflammatory cytokines, such as interferon gamma (IFN-γ) and tumor necrosis factor alpha, and produce proinflammatory cytokines for eliminating pathogens, tumor cells, and damaged tissues. In contrast, M2 macrophages are activated by cytokines, such as IL-4, IL-13, and IL-10, thereby producing anti-inflammatory cytokines and growth factors that are involved in tissue repair, wound healing, and immune regulation^7^. Cytokines released from M1 and M2 macrophages determine the activation profiles of helper T cells (Th), thereby making macrophage activation crucial for regulating adaptive immunity, including the Th1/Th2 balance^5^.

Glycolysis is a critical metabolic pathway for inducing M1 polarization^8–10^. When macrophages are polarized towards the M1 phenotype, the expression of glycolytic enzymes increases to enhance glucose uptake^8^. Conversely, inhibition of glycolysis suppresses inflammatory response, indicating that glycolytic activity is a key determinant of macrophage polarization.

Macrophage activation profiles vary among mouse strains. For instance, C57BL/6 (B6) and DBA/2 mice produce higher levels of IFN-γ and lower levels of IL-4 in response to lipopolysaccharide (LPS) than those produced by BALB/c mice^11^. BALB/c mice are more susceptible than B6 and DBA/2 mice to *Leishmania* infection^12^. These differences in immune profiles and responses can be explained by variations in the genomic backgrounds of mouse subspecies^13^. In fact, 56.7 million unique SNPs and 8.8 million unique indels have been identified from cross-sectional analysis of 17 inbred strains of laboratory mice^14,15^. *Mus musculus molossinus*, a subspecies derived from Japanese wild mice, has a genetic background distinct from that of *Mus musculus domesticus* that includes the commonly used reference strain B6^16^. Our previous analysis has revealed substantial genetic diversity between *Mus musculus domesticus* (B6) and *Mus musculus molossinus* including JF1. Specifically, 13.9 million SNPs and more than one million indels have been identified between B6 and JF1^17,18^. These extensive polymorphisms enable the detection of strain-specific allelic expression, facilitating the understanding of differential gene expression and its contribution to phenotypic variation among subspecies.

Individual differences also exist in human immune activation, and hyperactivation of the immune system can lead to immune diseases such as rheumatoid arthritis and allergies. Furthermore, immune profiles are linked to the onset of diseases such as diabetes, cancer, and cardiac disorders. Therefore, genetic variations that influence polarized activation of immune cells are promising for modeling human diseases caused by immune dysregulation. In this study, we compared the cellular phenotypes of primary macrophages between B6 and JF1 mice and observed distinct differences in their activation profiles. RNA expression analysis in reciprocal cross F1 hybrids has been widely used to distinguish allelic gene expression including imprinted genes^19–22^. Using F1 hybrids between B6 and JF1, we examined strain-specific differences in allele expression for revealing the molecular mechanisms of macrophage polarization driven by genetic factors.

## Results

### JF1 macrophages exhibit suppressed immune phenotype

**Fig. 1:**
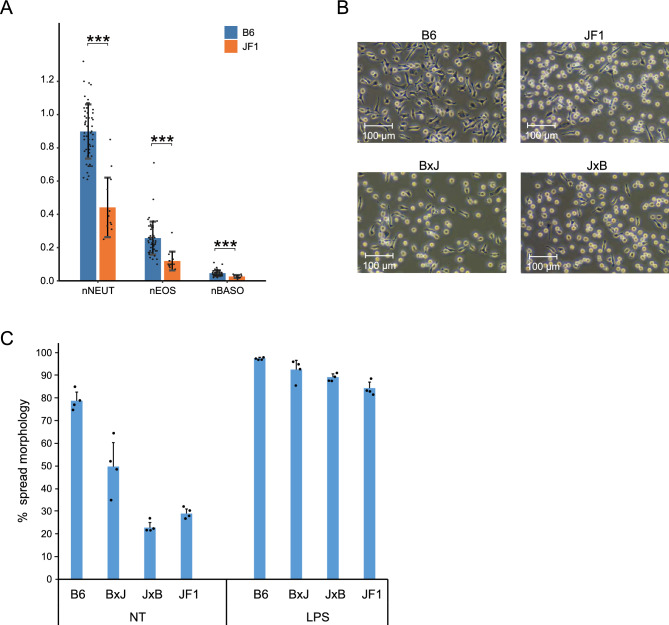
JF1 exhibits altered phenotype of macrophage activation. (**A**) Differences in populations of immune cells in peripheral blood between B6 and JF1 Each dot represents an individual mouse (B6, n = 56; JF1, n = 13). Bars indicate mean ± SD, and p values were calculated by Welch’s t-test. ***p < 0.001. (**B**) Morphological differences of macrophages between B6, JF1, and their F1 hybrid mice. Scale bar, 100 μm. (**C**) The population of spread morphology in peritoneal macrophages collected through peritoneal washing. Each dot represents an individual mouse (n = 4 per group), and error bars indicate the mean ± SD.

To compare the immune profiles between B6 and JF1 mice, we analyzed the white blood cell populations in peripheral blood. Both the absolute number and cellular fraction were significantly decreased in JF1 compared to those in B6, suggesting reduced activation of immune response in JF1 (Fig. 1A; Supplementary Fig. S1A). Next, we collected and observed tissue-resident macrophages from the peritoneal cavities of B6 and JF1 mice. Peritoneal macrophages collected from JF1 predominantly exhibited round morphology, indicating a suppressed inflammatory phenotype (Figs. 1B, C; Supplementary Fig. S1B). These results suggest that the degree of inflammatory activation in macrophages is lower in JF1 mice than in B6 mice.

### M1 activation is suppressed in JF1 macrophages

**Fig. 2:**
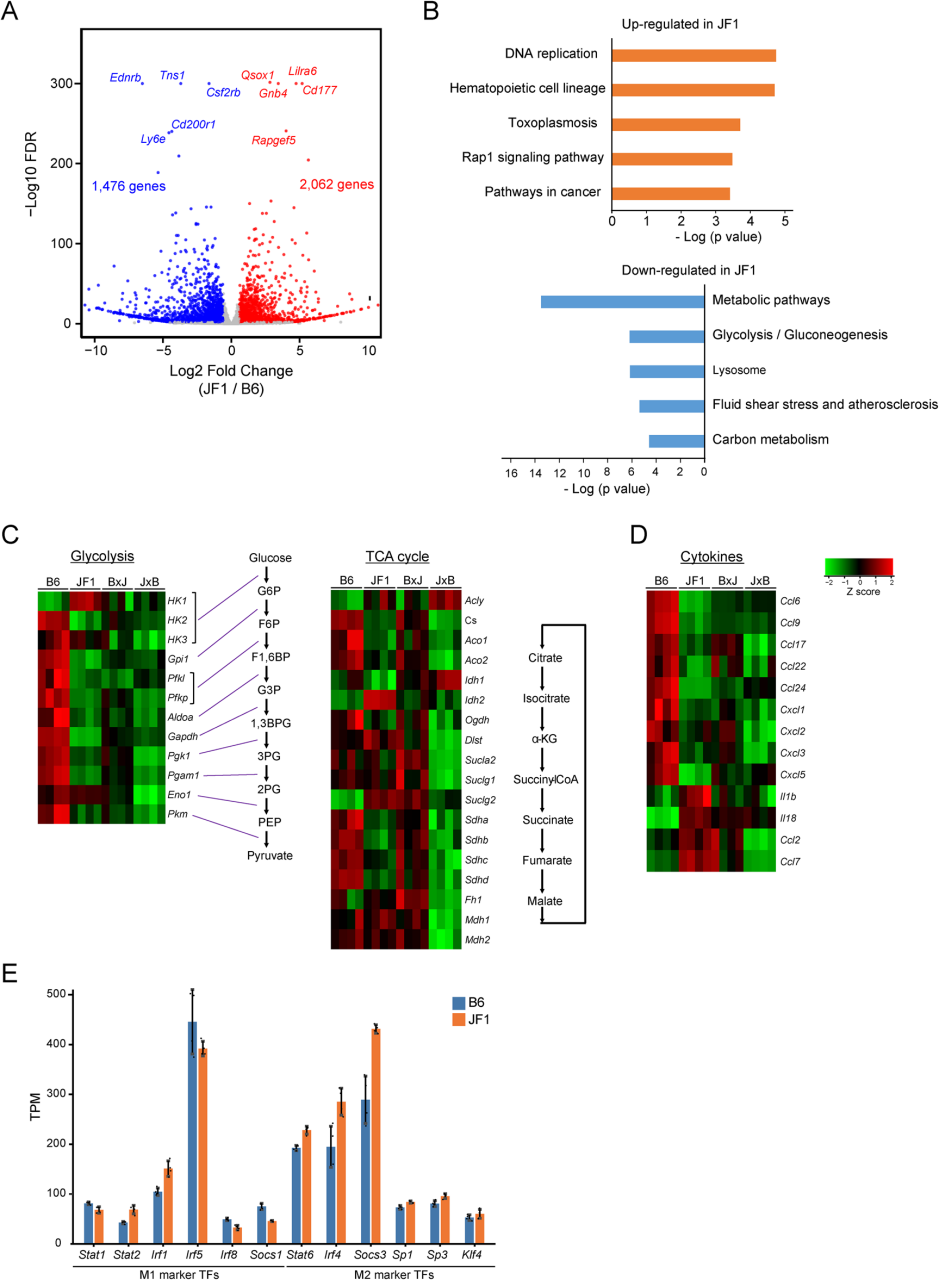
Transcriptomic divergence of macrophage polarization in B6 and JF1 mice. (**A**) Volcano plot of RNA-Seq in B6 vs. JF1 macrophages. Expression profile of macrophage was analyzed by RNA-seq with red and blue indicating significantly upregulated genes in JF1 and B6, respectively (|log2FC|> 1.5, FDR < 0.01). Data are the average of four biologically independent samples. (**B**) KEGG pathway analysis for upregulated or downregulated genes of JF1. P values were calculated by Fisher’s Exact test. (**C**) Expression of genes involved in glycolysis (left) and TCA cycle (right). The color scale indicates normalized level of gene expression in B6, JF1, and F1 hybrid mice and each row represents individual mouse. (**D**) Expression of genes encoding representative cytokines. The color scale indicates normalized level of gene expression in B6, JF1, and F1 hybrid mice and each row represents individual mouse. (**E**) Differential expression profile of M1/M2-marker TF genes between B6 and JF1. TPM values from RNA-seq data were used to compare the expression levels of representative transcription factors associated with M1 and M2 macrophage polarization. Error bars represent mean ± SD, and each dot represents an individual mouse (n = 4 per group).

To investigate the activation of altered immune pathway in mouse subspecies, RNA-Seq analysis was performed using peritoneal macrophages derived from B6, JF1, and reciprocal-cross F1 hybrids. A comparison of gene expression profiles revealed that 2,062 genes were upregulated, and 1,476 genes were downregulated in JF1 macrophages compared to those B6 macrophages (Fig. 2A; Supplementary Table 1). Reactome analysis suggested that the set of genes upregulated in JF1 macrophages was enriched for terms related to “DNA replication” and “Hematopoietic cell lineage” while the downregulated genes were significantly enriched for terms related to “Metabolic pathway” and “Glycolysis” and (Fig. 2B). Compared to that in B6 macrophages, the expression of key glycolytic enzyme genes, except for *Hk1, Hk3*, and *Eno1* decreased in JF1 and F1 hybrid macrophages (Fig. 2C). Conversely, the expressions of tricarboxylic acid (TCA) cycle-related genes were comparable across the strains, with some being upregulated in JF1 macrophages. Given that intracellular glycolytic activity determines macrophage polarization, cytokine expression patterns were analyzed. Compared to that in B6, the expression of chemokines encoded by genes, including *Ccl6*, *Ccl9*, and *Ccl17*, decreased, whereas the expression of cytokines encoded by genes, including *Il1b* and *Il18*, increased in JF1 macrophages (Fig. 2D). Furthermore, the expression of transcription factors promoting M1 activation generally decreased, whereas those promoting M2 activation increased in JF1 compared to those in B6 (Fig. 2E). These data indicate that the distinct profiles of polarized activation in B6 and JF1 macrophages are regulated at the transcriptional level.

### Downregulation of glycolysis in JF1 macrophages revealed by allele-specific expression analysis

**Fig. 3:**
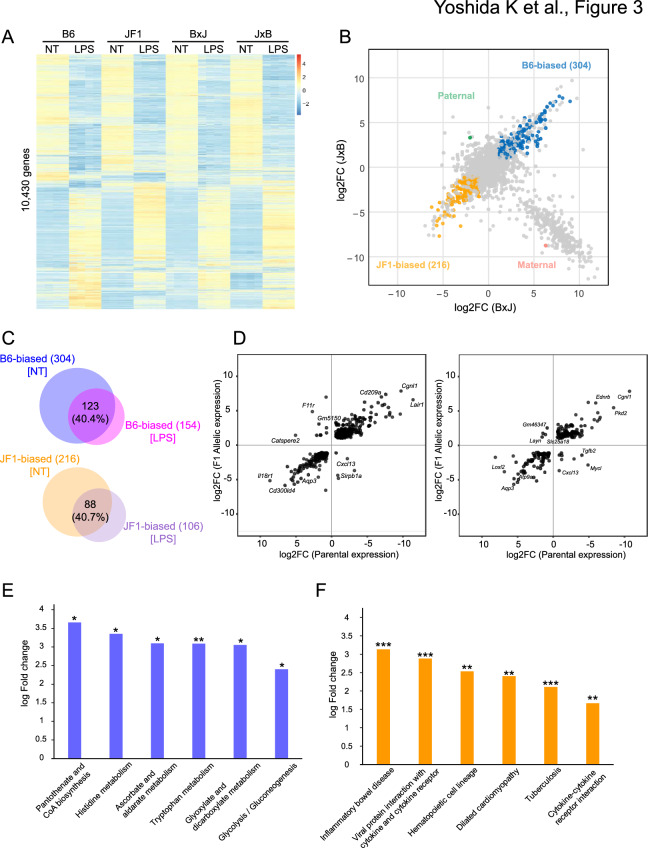
Strain-specific biased expression patterns in macrophages. (**A**) Heatmap visualization of RNA-seq data. Gene expression profiles across four mouse strains (B6, JF1, and their reciprocal F1 hybrids) are shown in response to LPS stimulation. (**B**) Scatter plot of *cis*-eQTL analysis based on log2 fold changes in B × J and J × B mice. Genes were categorized as B6-biased (blue), JF1-biased (orange), maternal (red), or paternal (green) based on the statistical criteria (|log2FC|> 1 and FDR < 0.05). (**C**) Comparison of B6-biased genes (top) and JF1-biased genes (bottom) between the untreated and LPS-treated groups. (**D**) Scatter plots comparing gene expression ratios between parental strains and reciprocal F1 hybrids under untreated (left) and LPS-treated (right) conditions. The x-axis shows the log2FC between B6 and JF1, and the y-axis shows the log2 allelic expression ratio (B6 allele vs. JF1 allele) in F1 hybrids. (**E**, **F**) KEGG Pathway analysis of B6-biased genes (E) and JF1-biased genes (F). Statistical significance was assessed using Fisher’s exact test. Asterisks indicate significance levels: p < 0.05 (*), p < 0.01 (**), and p < 0.001 (***).

The differential expression of immune and metabolic genes can be attributed to genetic variations between B6 and JF1 mice. To investigate how strain differences influence gene expression responses to immune stimulation, we performed RNA-seq analysis of macrophages with and without LPS treatment (Fig. 3A; Supplementary Fig. S2A-C). In JF1 macrophages, 1,861 genes were upregulated and 2,061 were downregulated, whereas B6 showed a more limited response with 1,581 upregulated and 1,696 downregulated genes. A large proportion of LPS-responsive genes were shared across all four strains, suggesting a conserved core transcriptional program for immune responses. Whereas each of the four strains harbored a substantial number of strain-specific differentially expressed genes (DEGs) (Supplementary Fig. 2D), These results indicated that the transcriptional response to LPS was not only shaped by genetic background but also partially restructured in F1 hybrids, likely reflecting non-additive effects of parental genomes.

To identify the genes with expression affected by strain-specific variants, we performed *cis*- expression quantitative trait locus (eQTL) analysis that distinguishes allele-specific expression (ASE) for evaluating the impact of *cis*-regulatory elements. First, using the same RNA-seq datasets, maternal and paternal imprinted genes were identified from macrophages derived from reciprocal-cross F1 hybrids by applying an allele-separated pseudogenome^23^ (Fig. 3B, Supplementary Table 2). Among the 91 maternally biased genes, 90 mapped to the X chromosome and likely reflect sex-linked expression in male F1 hybrids rather than imprinting; the remaining autosomal gene was the known imprinted gene, *Igf2r.* We also detected the known imprinted genes, *Zrsr1,* as paternally expressed^24,25^, confirming detectability of parent-of-origin effects when genes are sufficiently expressed. However, few autosomal imprinted genes were detected, possibly reflecting limited expression of candidate loci under our conditions and the use of conservative filters that may reduce sensitivity.

The *cis*-eQTL analysis identified 304 B6-biased genes with relatively high expression from the B6 allele, and 216 JF1-biased genes with relatively high expression from the JF1 allele (Figs. 3B, S3A). Following LPS treatment, 154 B6-biased and 106 JF1-biased genes were detected (Supplementary Fig. 3B). Approximately 40% of the initially identified biased genes maintained their allelic preference after stimulation, suggesting that a subset of *cis*-regulatory effects remains stable under inflammatory conditions (Fig. 3C). To further investigate the contribution of *cis* effects on gene expression, we compared the expression ratios between B6 and JF1 in the parental strains with the allelic expression ratios in reciprocal F1 hybrids under LPS-treated conditions (Fig. 3D). Most genes aligned along the diagonal in both untreated and treated conditions, indicating that strain-specific expression differences are largely attributable to *cis*-acting variants. Since LPS stimulation did not lead to a marked horizontal spread of data points, only a limited number of genes appear to be primarily regulated by *trans*-acting factors even under immune-activated conditions.

Kyoto Encyclopedia of Genes and Genomes (KEGG) pathway analysis of strain-specific biased genes revealed enrichment in glycolysis-related pathways (Figs. 3E, 3 F, S3C, S3D), suggesting that *cis*-acting genomic variants unique impacted the regulation of these genes. Cytokine-coding genes, such as *Ccl3*, and *Ccl6*, were highly expressed from the B6 alleles in F1 macrophages, whereas *Ccl2*, *Ccl7*, *Cxcl13*, and *Il18* (*Il18r1*) were expressed from the JF1 alleles. The increased expression of genes involved in viral response suggests differences in pathogen responsiveness between mouse subspecies. B6-derived macrophages were more responsive to LPS than JF1-derived macrophages at the cellular and gene-expression levels, implying an enhanced adaptability to pathogen infections. Our findings are consistent with previously reported involvement of *cis*-regulatory elements in LPS responsiveness among mouse strains^26^. Among the major histocompatibility complex (MHC) genes, *H2-EB1* and *H2-M3* were highly expressed from the B6 alleles, whereas *H2-Oa* was biased toward the JF1 allele. The diversity of MHC genes across subspecies plays crucial roles in antigen presentation and regulation of immune response^27,28^. Our F1 expression analysis suggests that polymorphisms in *cis*-regulatory elements within the MHC region contribute to differences in strain-specific expression. Strain-specific upregulation of ribosomal protein-coding genes, such as *Rpl23*, *Rpl28*, and *Rpl35a* from the B6 allele and *Rpl18a* from the JF1 allele, suggests distinct potential for protein synthesis in macrophages between two strains. This enhanced protein synthesis, along with the upregulation of immune-related genes, may contribute to macrophage activation. Notably, the genes expressing glycolytic enzymes were highly ranked among the DEGs in both the F1 hybrids and parental strains. Given that cytokine expression is linked to glycolytic activity^9^, glycolytic activity may influence functional properties of macrophages.

### Influence of structural variants on strain-specific gene expression regulation

**Fig. 4:**
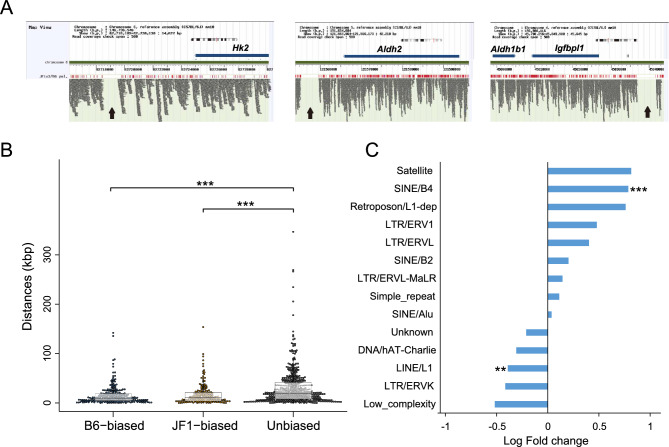
Association between SVs and differential gene expression. (**A**) Mapping profiles of JF1-derived short reads aligned to the B6 genome. Screenshots from the MoG + genome browser show short-read alignments from the JF1 genome assembly (JF1agv1.0) mapped to the B6 reference genome (GRCm38/mm10). Representative strain-specific biased genes, such as *Hk2*, *Aldh2*, and *Igfbpl1,* show clear short-read coverage gaps (arrows). (**B**) Positional relationships between DEGs and gaps. The distances from the TSSs of B6-biased, JF1-biased, and unbiased genes to the nearest gaps. Statistical significance was assessed using Wilcoxon rank-sum test, p < 0.001 (***). (**C**) The log2 fold change in the frequency of repeat classes within genomic regions surrounding B6-biased genes, compared to the genome-wide background. Enrichment analysis was performed for each repeat class, and statistically significant differences are indicated (***p < 0.001, **p < 0.01; Fisher’s exact test).

The differences in allelic expression between B6 and JF1 macrophages were assumed to be influenced by *cis*-regulatory variants. We specifically focused on structural variants (SVs), such as relatively large insertions and deletions, which may have a significant impact on strain-specific gene expression. To explore potential signatures of SVs, we examined the positions of unresolved assembly gaps in the JF1 genome. Through comparison with the B6 reference genome, a total of 154,808 long indels > 200 bp were identified in the genomic sequence of JF1 (Supplementary Table 3). Notably, these gaps were found around some B6-biased genes, including *Hk2*, whose expression decreased in the JF1 allele (Fig. 4A). To further investigate this, we analyzed the positional correlation between biased genes and these gaps by calculating the distance between the gaps and transcription start sites (TSS) of strain-specific biased genes. The gaps were significantly closer to the TSSs of biased genes than to allelically unbiased genes, indicating that these assembly gaps, as potential SVs, may affect local *cis*-regulatory activity (Fig. 4B).

To examine the genomic content of these gaps, we referred to the long read-based genome assembly (JF1agv1.1), which enabled sequence resolution across previously unresolved regions. This analysis revealed that some of the gap regions contained repetitive sequences. The distribution of classes of repetitive sequences around B6-biased genes in the JF1 genome revealed a significant enrichment of SINE/B4 (Fig. 4C). The presence of SINE/B4 sequences near JF1 alleles may be associated with reduced expression. In contrast, LINE/L1 elements were significantly underrepresented around genes that showed relatively lower expression in the JF1 allele (Fig. 4C), suggesting that the presence of LINE/L1 in the JF1 genome may contribute to enhanced expression of the alleles. These opposing trends imply that distinct classes of repetitive sequences may differentially influence gene expression, potentially contributing to differences in macrophage polarization between the B6 and JF1 strains.

## Discussion

Disease models of experimental animals are invaluable for evaluating molecular pathology, disease prevention, and therapeutic drugs. The selection of an appropriate animal model and its genetic background is crucial for these studies. For example, M1 macrophage activation can promote the progression of rheumatoid arthritis by activating osteoclasts through the secretion of proinflammatory cytokines^29^. Conversely, M2 macrophage activation, which is associated with homeostasis, injury, and tissue repair, may protect against renal diseases^30^. In this study, cellular observations and transcriptomic analysis indicated that peritoneal macrophages from JF1 mice exhibited altered polarization of macrophage activation compared to that of the standard reference strain B6. Macrophage polarization towards M1 or M2 is correlated with the onset of various diseases.

The mouse subspecies consist of *Mus musculus domesticus*, *Mus musculus musculus*, and *Mus musculus castaneus*. Notably, *Mus musculus molossinus* is a hybrid species derived from interbreeding of *musculus* and *castaneus*. Genetic diversity among different mouse subspecies provides an important resource for understanding differential gene expression and its impact on phenotypic variation, including immune cell function^11–13^. Significant genomic variations, including SNPs, indels, and SVs, play critical roles in regulating gene expression across different tissues and contribute to distinct phenotypic outcomes. Strain-specific expression plays a crucial role in determining immune cell phenotypes^31^.

Our gene expression analysis of macrophages derived from F1 hybrids revealed distinct expression bias associated with the parental strains. KEGG pathway analysis identified four groups of B6-biased genes: those encoding ribosomal proteins, MHC antigens, innate immunity-related cytokines, and glycolytic enzymes. Our findings imply that *cis*-regulatory polymorphisms in immune and metabolic pathways may influence the transcriptional profiles and functions of B6 and JF1 macrophages. However, the functional relevance of these transcriptional patterns remains unclear, and their impact on macrophage phenotypes awaits further investigation.

Repetitive sequences in the genome are broadly categorized into tandem and interspersed repeats, each contributing to the chromosomal structure and function^32–34^. Tandem repeats are primarily concentrated in regions such as the centromeres and telomeres. Interspersed repeats, including transposable elements such as LINEs, SINEs, and endogenous retroviruses, are scattered across introns and intergenic regions and often affect gene expression by modifying local transcriptional activity^35,36^. In various organisms, from yeast to vertebrates, these groups of repetitive sequences maintain their heterochromatin structures and produce small RNAs via the RNA interference pathway^37,38^. Furthermore, these heterochromatin structures can spread around genomic regions and suppress the transcriptional activity of neighboring genes. SVs, including transposable elements, can affect genome organization and gene expression, suggesting that they play a significant role in phenotypic differences across mouse subspecies^36^. SVs often remain undetected through short-read sequencing because of its limitations in mapping against the reference genome. Our gap-based approach treated unresolved short-read assembly gaps as proxies for SVs. Because this is an indirect measure, it cannot resolve breakpoints and may capture assembly artifacts. Within this framework, our positional analysis indicated an enrichment of putative SVs near genes exhibiting ASE. Furthermore, some groups of repetitive sequences, including LINE/L1 and SINE/B4, were enriched in these indels. These results suggest that many JF1 indels function as gene regulators via repetitive sequences, and that JF1 may be useful for modeling diseases characterized by the activation of M2 macrophages.

The expression of a group of glycolytic enzymes, including hexokinase 2 (HK2) encoded by *Hk2*, decreased in JF1 macrophages. A positive correlation between glycolytic activity and macrophage M1 polarization has recently been indicated, thereby suggesting that differential expression of inflammatory cytokine genes between B6 and JF1 can be explained by differences in glycolytic activities^39^. In fact, the expression of transcription factors that promote M1 polarization decreased in JF1 macrophages. HK2 functions as a rate-limiting enzyme in glycolysis, catalyzing the first committed and irreversible step by phosphorylating glucose to glucose-6-phosphate. This reaction serves as a metabolic switch, determining whether glucose will be utilized for energy production. Notably, inhibition of HK2 has been reported to suppress sepsis shock in mouse model, indicating an enhanced role of *Hk2* in inflammatory responses^40^. SVs near glycolytic genes, such as *Hk2*, may contribute to the phenotypic diversity of macrophages among mouse subspecies.

In addition to *Hk2*, other B6-biased genes, such as *Aldh2* and *Aldh1b1* were identified through ASE analysis. In contrast to *Hk2*, upregulation of these two genes has been reported to promote M2 macrophage activation^41,42^. The distinct patterns of subspecies-specific biased genes highlight the intricate genetic regulatory mechanisms underlying the phenotypic diversity of B6 and JF1 macrophages. Consistent with the parental strain differences, the direction and magnitude of allelic expression in F1 hybrids closely reflect those observed in the parental strains. This indicates that *cis*-regulatory elements substantially contribute to strain-specific gene expression in macrophages. Furthermore, a significant correlation was noticed between the genomic locations of biased genes and SVs including transposable elements. This suggests that SVs may influence the transcriptional activity of nearby genes, thereby contributing to subspecies-specific gene expression profiles in mouse macrophages. Notably, SINE elements such as SINE/B4 were significantly enriched around B6-biased genes, suggesting that insertions of SINEs in the JF1 genome may suppress gene expression from the JF1 allele.

In contrast, the expression profiles of genes responding to external stimuli (LPS) were relatively similar between B6 and JF1 macrophages, suggesting that fundamental immune responses are largely conserved across strains. Given the magnitude of allelic imbalance changed following LPS exposure, environmental cues appear to modulate the effects of *cis*-regulatory variants. The persistence of allelic bias along the same axis as parental strain differences supports a model of gene-by-environment interactions where LPS stimulation fine-tunes *cis* effects through *trans*-acting regulators. Although some exceptions were observed, such as genes exhibiting expression changes unique to F1 hybrids or those strongly influenced by *trans*-acting factors, the majority of genes displayed parallel shifts in total and allelic expression. Thus, comparison of allele-specific and total expression highlights the predominance of *cis*-regulatory polymorphisms, whose impact can be modulated by environmental stimuli such as LPS. Allele-specific expression analysis using F1 hybrids is a valuable system for evaluating both fundamental cellular signal responses and strain-specific modulation of gene expression regulated through *cis*-polymorphisms in parallel. The presence of *cis*-variants and their effects on gene expression provide insights into how genetic diversity can lead to unique phenotypic modifications across mouse strains.

## Methods

### Mice

Male wild-type C57BL/6 and JF1/Ms mice (7–8 weeks old) were used. C57BL/6 mice were purchased from Japan CREA, and JF1/Ms mice were obtained from the RIKEN BioResource Center (RBRC00639). Mice were housed under standard conditions (temperature: 25 °C, humidity: 55%, 12 h/12 h light/dark cycle) with ad libitum access to food and water. For sample collection, mice were euthanized by cervical dislocation as an appropriate method of sacrifice, and no anesthesia was administered. All animal experiments were approved by the Institutional Animal Care and Use Committee of RIKEN Tsukuba Branch (Approval number: T2024-EP008) and were conducted in accordance with the institutional guidelines, which adhere to national regulations for animal welfare. This study follows the principles of the ARRIVE guidelines where applicable, ensuring transparent and comprehensive reporting of animal research. The sample size per group was determined based on previous experience ensuring statistical significance. The investigators were not blinded to the experimental conditions, and no randomization or exclusion of data points was performed.

### Cell culture

Peritoneal macrophages were isolated from mice as previously described^43^. Peritoneal macrophages were collected by peritoneal washing, without injection of thioglycollate, and were incubated in Macrophage-SFM (#12,065,074; Life Technologies) containing 1 ng/mL GM-CSM (#576,302; BioLegend), 100 U/mL penicillin, 100 μg/mL streptomycin in 60-mm dishes. After incubation for 3 h, the dish surfaces were washed once with PBS, followed by three washes without pipetting, to remove non-adherent cells. After an overnight incubation, the dish surfaces were washed twice with phosphate-buffered saline (PBS), followed by incubation for an additional 3 h, after which adherent cells were used as peritoneal macrophages. To observe macrophage activation, cells were treated with LPS (100 ng/mL; *E. coli* O111:B4, #L2630; Sigma-Aldrich) for 3 h. Statistical significance in morphological differences with or without of LPS stimulation was evaluated using the two-tailed Welch’s t-test with p-value < 0.05. Peripheral blood cell populations were analyzed using the standardized pipeline of the Japan Mouse Clinic at the Riken BRC. Statistical differences between B6 and JF1 strains were evaluated using the two-tailed Welch’s t-test with p-values < 0.05.

### RNA-Seq analysis

RNA was isolated from macrophages (median lobe) of 8-week-old individual mouse using TRIzol (#15,596,018; Thermo Fisher Scientific) reagent, according to the manufacturer’s instructions (Supplementary Fig. S4A, B). Total RNA (500 ng) was converted into RNA-Seq libraries using rRNA Depletion Kit (E6310; New England Biolabs) and Next Ultra Directional RNA Library Prep Kit (E7420; New England Biolabs). Sequencing was performed using a NextSeq500 system (Illumina) to obtain paired-end 2 × 36 base reads. To enable ASE analysis, a pseudogenome was constructed by combining the B6 reference genome (GRCm38/mm10) with JF1 genome (JF1agv1.1) derived from the MOG + database at the RIKEN BRC^18^. Only primary chromosomes (chr1–19, X, Y, M) were retained and minor contigs were excluded. Chromosome names were suffixed with _B6 or _JF1 and the two genome assemblies were concatenated into a single FASTA file. Matched Gene annotations were processed by lifting over the B6 GFF3 file to the JF1 genome using Liftoff, followed by conversion to GTF format using gffread^44^. After quality control with FastQC, RNA-seq reads from B6, JF1, and reciprocal F1 hybrids were mapped to the pseudogenome using CLC Genomics Workbench v.10.1.1 (QIAGEN), following the official user manual (version 6.5). Because the software is proprietary, we report the user-level mapping parameters to ensure reproducibility: mismatch cost = 2, insertion cost = 3, deletion cost = 3, length fraction = 0.8, and similarity fraction = 0.8. These settings moderately penalize mismatches and indels while requiring ≥ 80% read coverage and identity, ensuring a balance between sensitivity and specificity for high-confidence alignments suitable for downstream analysis (Supplementary Fig. S4C; Supplementary Table 4). Read counts and TPM values were calculated and used for downstream analysis using R. Differential gene expression was evaluated using DESeq2 in R with significance defined as false discovery rate (FDR) < 0.01.

### *Cis*-eQTL analysis

Approximately 17% of allele-specific entries with potential mismapping (a minor allele count exceeded an expected allele with < 10 reads) were excluded based on parental strain expression patterns. ASE analysis was performed using read count data from reciprocal F1 hybrids only for genes with more than 10 total reads. A beta-binomial model (glmmTMB) was applied to detect allelic imbalance per gene and per cross^45^. Genes were categorized as B6-biased, JF1-biased, maternal, paternal, or unbiased based on reciprocal log2 fold changes (|log2FC|≥ 1) and FDR (< 0.05). Functional analysis of allelic imbalanced genes was performed using DAVID functional annotation system^46,47^, which incorporates multiple databases including KEGG^48–50^. Pathway names in bar charts were based on the KEGG database.

### Gap-based structural variant prediction

We treated unresolved assembly gaps in JF1agv1.0 (short-read assembly) as proxies for loci likely to harbor SVs. Coordinates of the gap regions were obtained as a BED file. Distances between TSSs of expressed genes were calculated using ClosestBed (v2.31.1). The distance data of B6-biased (n = 256), JF1-biased (n = 192), and representative unbiased genes (n = 498) were visualized using the ggbeeswarm R package (version 0.7.2), available at https://cran.r-project.org/web/packages/ggbeeswarm/index.html. Statistical significance of distance differences was assessed using Wilcoxon rank-sum test (p < 0.001).

To investigate potential SVs within previously unresolved genomic gap regions, we referred to the current JF1agv1.1 genome assembly, which was constructed using long-read sequencing technology. This updated assembly enabled us to identify SVs embedded within these regions. Based on this improved reference, we classified the SVs in each gap region. For each class of repeat elements (*e.g*., SINEs, LINEs, LTRs), we calculated their frequency, and then enrichment was quantified as the log2 fold change of the repeat frequency in the B6-biased gene set compared to the genome-wide background.

### Quantification and statistical analysis

All experiments were performed using independent biological replicates. Statistical analyses were performed using R (version 4.3.3). For pairwise comparisons, two-tailed unpaired Welch’s t-tests or Wilcoxon rank-sum tests were used based on data distribution. Details of specific statistical tests and sample sizes are provided in figure legends and Methods. Data are presented as mean ± standard deviation (SD) unless otherwise noted.

## Supplementary Information

Supplementary Information 1.

Supplementary Information 2.

Supplementary Information 3.

Supplementary Information 4.

Supplementary Information 5.

Supplementary Information 6. Supplementary Tables Table S1. TPM values of gene expression in peritoneal macrophages from four mouse strains (B6, JF1, BxJ, and JxB) under non-treated and LPS-stimulated conditions. This table corresponds to the gene expression analysis shown in Figure 2. Table S2. Log2FC of allele-specific gene expression in reciprocal F1 hybrids (BxJ and JxB) used for cis-eQTL analysis in Figures 3 and S3. Table S3. Genomic coordinates of gaps in the JF1 genome used in Figure 4. The table lists the chromosome, start and end positions, gap names, sizes, and strand orientation. Table S4. Mapping summary of RNA-Seq reads to the hybrid genome between B6 and JF1

## Data Availability

All sequencing data generated using the next-generation sequencer were deposited in the DDBJ Sequence Read Archive (DRA) under the accession number PRJDB18900.
