# Supplementary material for: Allele-specific gene expression in F1 hybrid mice reveals structural variants affecting macrophage characteristics
Source: Sci Rep. 2025 Oct 29;15:37846. doi: 10.1038/s41598-025-21643-w (PMC12572132; doi:10.1038/s41598-025-21643-w)
Supplement: Supplementary file 1 — Supplementary Information 1. [file 41598_2025_21643_MOESM1_ESM.pdf]

A

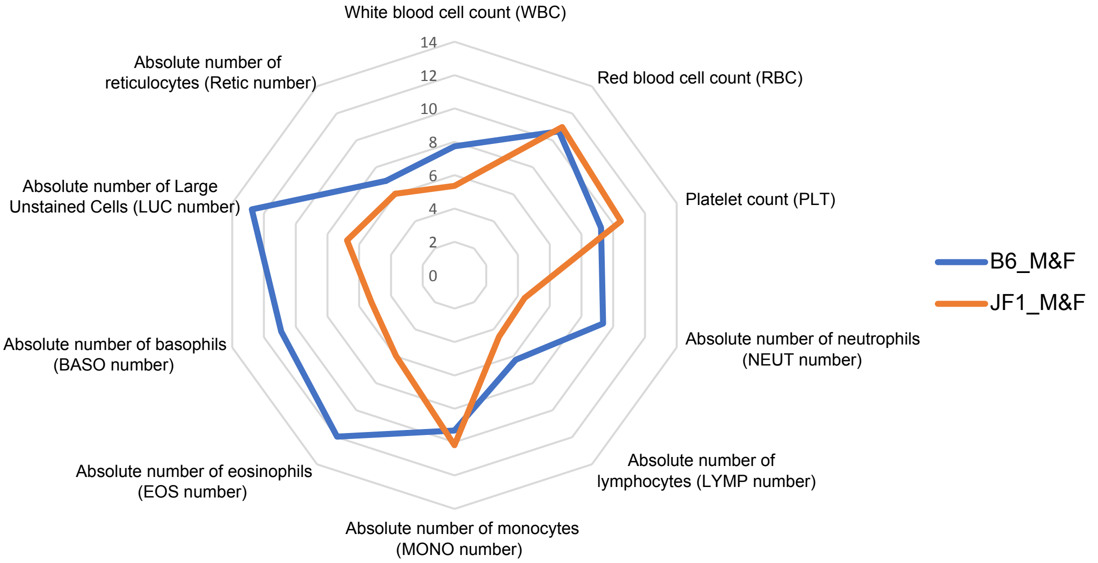

B

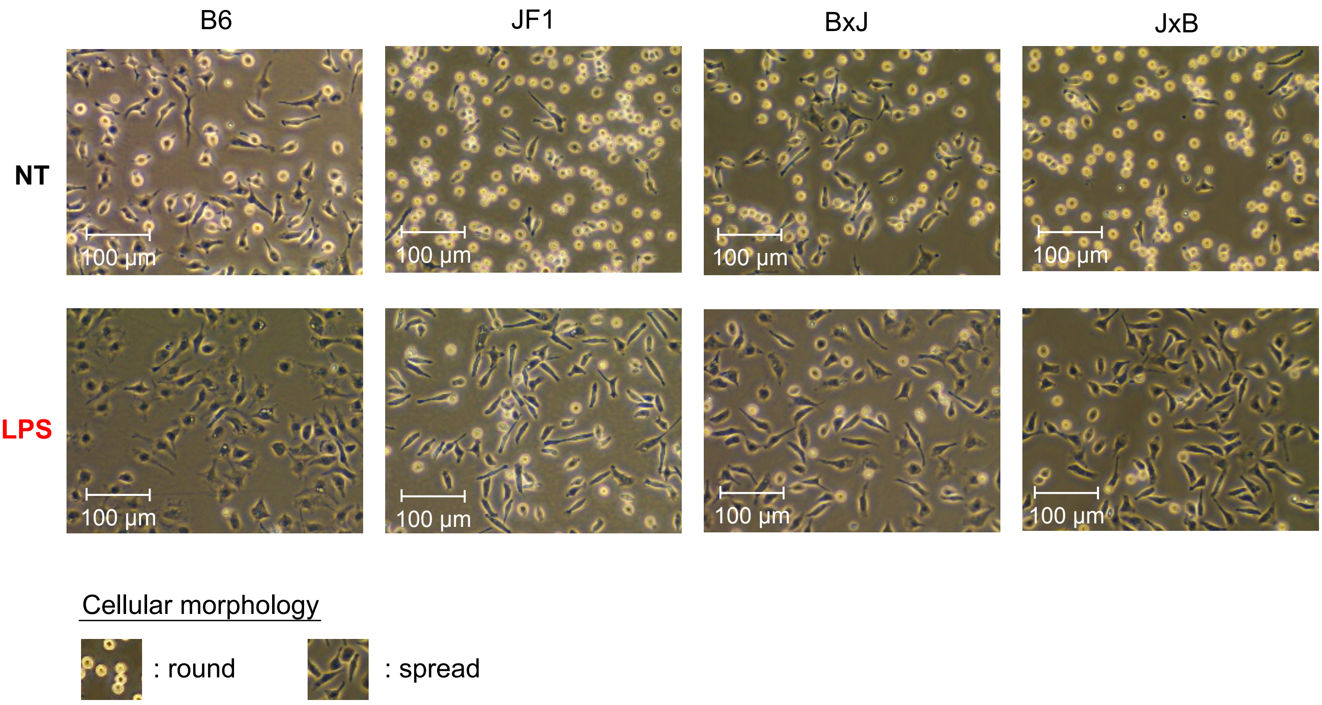

Supplementary Figure S1. Blood cell profiles and cellular morphology in B6 and JF1

(A) Quantification of blood cell profiles based on the cell counts of each cell type. Data is shown for male and female mice of B6 (blue) and JF1 (orange). (n = 56 mice in B6, n = 13 mice in JF1). The hematological analysis was performed at the Japan Mouse Clinic, RIKEN BRC (https://ja.brc.riken.jp/lab/jmc/mouse_clinic/).

(B) Representative cellular morphology under non-treated (NT) and LPS-stimulated conditions for B6, JF1, and reciprocal F1 hybrids (BxJ and JxB). Scale bars, 100 µm.

Yoshida K et al., Figure S2

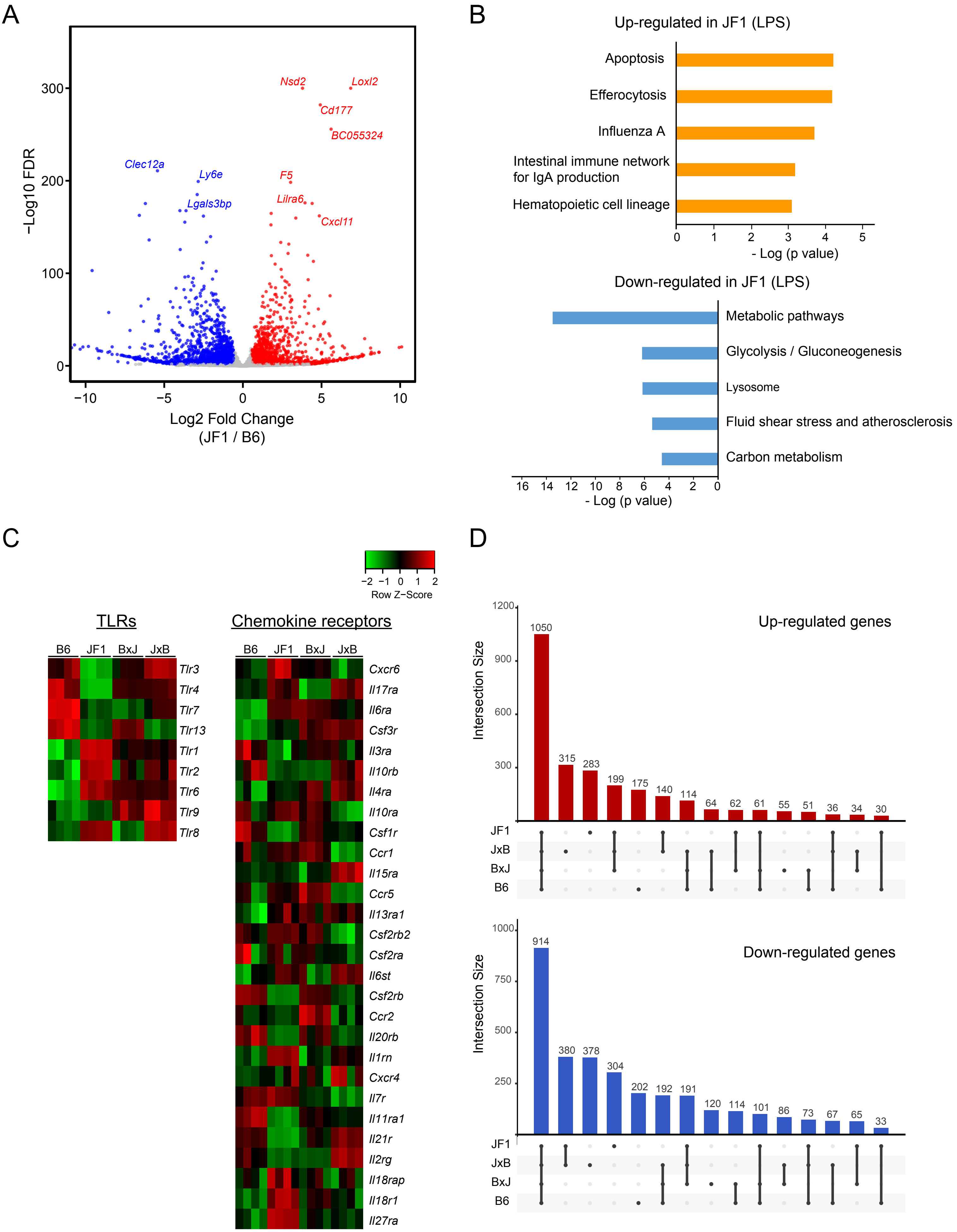

Supplementary Figure S2. Gene expression profiles in B6 and JF1 macrophages stimulated by LPS

(A)Volcano plot of RNA-Seq data showing differentially expressed genes in LPS-stimulated macrophages from B6 and JF1. Data represent the average of four biological replicates.

(B) KEGG Pathway enrichment analysis of upregulated and downregulated genes in JF1 mice under LPS stimulation. P values were calculated using Fisher's Exact test.

(C) Heatmap displaying the expression levels of genes encoding Toll-like receptors (TLR) and chemokine receptors in B6, JF1, and their reciprocal F1 hybrids macrophages after LPS stimulation.

(D) Upset plot of genes illustrating the overlap of upregulated (top) and downregulated (bottom) genes among B6, JF1, BxJ, and JxB macrophages after LPS stimulation.

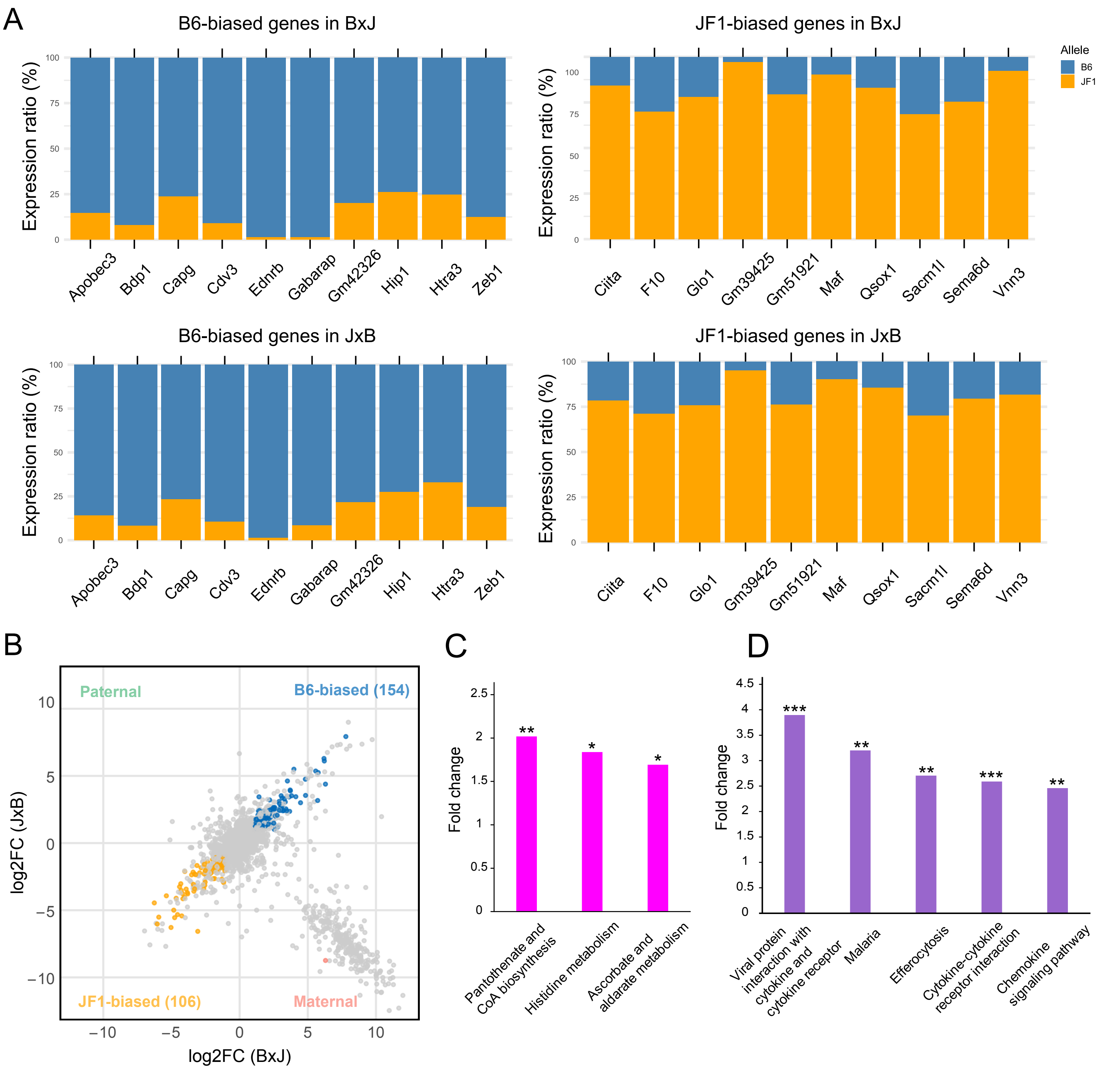

Supplementary Figure S3. Allelic expression in cis-eQTL analysis

(A) Allelic expression ratios of representative strain-specific biased genes in BxJ and JxB F1 hubrids.

(B) Scatter plot of cis-eQTL analysis based on log2 fold changes in B×J and J×B mice after LPS stimulation. Genes were categorized as B6-biased (blue), JF1-biased (orange), maternal (red), or paternal (green) based on the statistical criteria (|log2FC| >1 and FDR < 0.05).

(C, D) KEGG pathway analysis of B6-biased genes (C) and JF1-biased genes (D) under LPS stimulation. Statistical significance was assessed using Fisher's exact test. Asterisks indicate significance levels: $p < 0.05$ (*), $p < 0.01$ (**), and $p < 0.001$ (***).

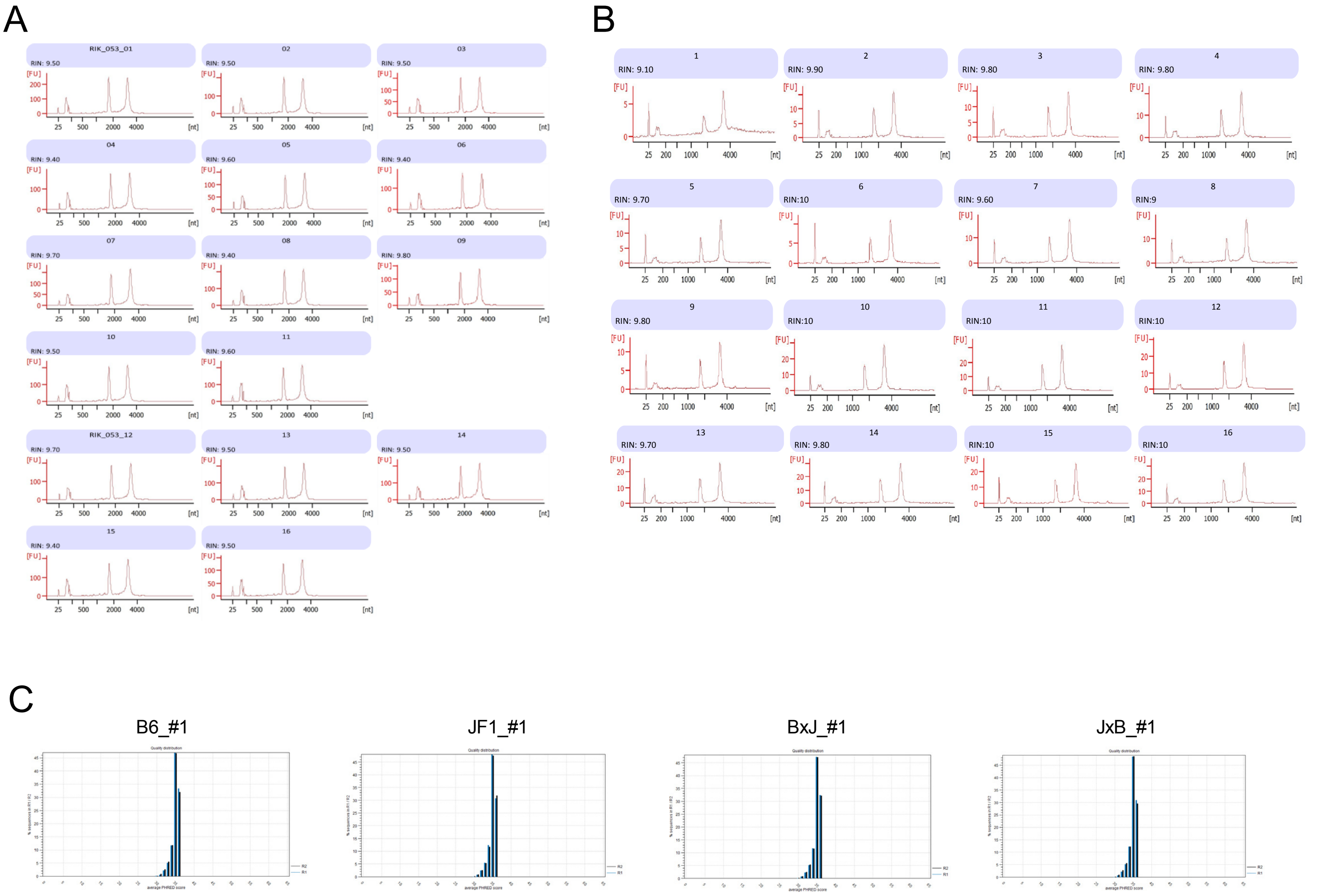

Supplementary Figure S4. Quality assessment of RNA-seq data

(A, B) Electropherogram profiles of total RNA from non-treated (A) and LPS-stimulated (B) macrophages. RNA integrity number (RIN) scores are indicated for each sample.

(C) Representative data of read distributions with Phred base quality scores.
