# Supplementary material for: Allele-specific gene expression in F1 hybrid mice reveals structural variants affecting macrophage characteristics
Source: Sci Rep. 2025 Oct 29;15:37846. doi: 10.1038/s41598-025-21643-w (PMC12572132; doi:10.1038/s41598-025-21643-w)
Supplement: Supplementary file 6 — Supplementary Information 6. Supplementary Tables Table S1. TPM values of gene expression in peritoneal macrophages from four mouse strains (B6, JF1, BxJ, and JxB) under non-treated and LPS-stimulated conditions. This table corresponds to the gene expression analysis shown in Figure 2. Table S2. Log2FC of allele-specific gene expression in reciprocal F1 hybrids (BxJ and JxB) used for cis-eQTL analysis in Figures 3 and S3. Table S3. Genomic coordinates of gaps in the JF1 genome used in Figure 4. The table lists the chromosome, start and end positions, gap names, sizes, and strand orientation. Table S4. Mapping summary of RNA-Seq reads to the hybrid genome between B6 and JF1 [file 41598_2025_21643_MOESM6_ESM.pdf]

**Supplementary Tables**

Table S1. TPM values of gene expression in peritoneal macrophages from four mouse strains (B6, JF1, BxJ, and JxB) under non-treated and LPS-stimulated conditions. This table corresponds to the gene expression analysis shown in Figure 2.

Table S2. Log2FC of allele-specific gene expression in reciprocal F1 hybrids (BxJ and JxB) used for cis-eQTL analysis in Figures 3 and S3.

Table S3. Genomic coordinates of gaps in the JF1 genome used in Figure 4. The table lists the chromosome, start and end positions, gap names, sizes, and strand orientation.

Table S4. Mapping summary of RNA-Seq reads to the hybrid genome between B6 and JF1.
